# Five‐Decade Mortality Trends in Ischemic Stroke in the United States: A CDC WONDER Analysis

**DOI:** 10.1002/brb3.71177

**Published:** 2025-12-31

**Authors:** Rameez Qasim, Laiba Muzammil, Bilal Qammar, Areej Dar, Laiba Sultan, Mubashir Raza, Umama Alam, Raheel Ahmed, Hasibullah Aminpoor, Muhammad Khalid Afridi, Saad Ahmed Waqas

**Affiliations:** ^1^ Department of Medicine Allama Iqbal Medical College Lahore Pakistan; ^2^ Department of Medicine Quaid‐e‐Azam Medical College Bahawalpur Pakistan; ^3^ Department of Medicine Shalamar Hospital Lahore Pakistan; ^4^ Department of Medicine Shaikh Khalifa Bin Zayed Al‐Nahyan Medical and Dental College Lahore Pakistan; ^5^ Department of Medicine Dow University of Health Sciences Karachi Pakistan; ^6^ Department of Medicine Baqai Medical University Karachi Pakistan; ^7^ Department of Medicine Khyber Medical College Peshawar Pakistan; ^8^ Department of Cardiology Newcastle Hospitals NHS Foundation Trust Newcastle Upon Tyne UK; ^9^ Faculty of Medicine Kabul University of Medical Sciences “Abu Ali Ibn Sina” Kabul Afghanistan

**Keywords:** CDC WONDER, epidemiology, mortality, racial disparities, ischemic stroke, United States

## Abstract

**Introduction:**

Ischemic stroke, comprising nearly 85% of all strokes, remains a leading cause of death and disability worldwide. Annually, about 795,000 individuals in the United States (US) experience a new or recurrent stroke. Despite advancements in diagnosis, treatment, and prevention, significant disparities and geographic variability persist, highlighting the need for targeted strategies to address these ongoing challenges.

**Methods:**

We analyzed US death certificate data from Centers for Disease Control and Prevention Wide‐ranging Online Data for Epidemiologic Research (1968‐2023) for ischemic stroke mortality using International Classification of Diseases (ICD‐8, ICD‐9, and ICD‐10) codes. Demographics included age, sex, and race/ethnicity. Crude and age‐adjusted mortality rates (AAMRs), annual percent change (APC), and average annual percent changes (AAPC) were calculated using joinpoint regression, and a *p*‐value less than 0.05 defined significance.

**Results:**

From 1968 to 2023, ischemic stroke mortality declined substantially, with 1,363,668 total deaths and the AAMR decreasing from 76.2 to 10.0 per 100,000 (AAPC: −3.59%). Early steep declines occurred from 1968 to 1982 and continued through 2014, followed by pronounced increases between 2014–2017 (APC 38.69) and 2017–2023 (APC 7.48). Men consistently exhibited higher AAMRs than women, with long‐term declines, yet both experienced recent upward trends. Racial disparities persisted, with Black adults declining from 85.9 to 14.2 (AAPC: −3.30%) and White from 75.5 to 9.8 (AAPC: −3.63%), but both showed reversals after 2014. Older adults (≥65 years) bore the greatest burden (AAPC: −3.91%), while younger age groups exhibited smaller reductions.

**Conclusion:**

Despite long‐term declines in mortality, recent increases and persistent disparities by age, sex, and race underscore the need for targeted prevention and equitable healthcare interventions in particularly high‐risk populations.

## Graphical abstract

This graphical abstract depicts long‐term trends in ischemic stroke mortality in the United States using CDC WONDER data. Over five decades, age‐adjusted mortality rates declined substantially, with the highest burden observed among older adults. Persistent disparities were evident by sex and race, with higher mortality among males and Black or African American individuals compared with other groups.

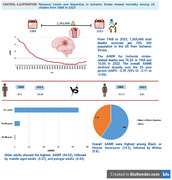

**Abbreviations**
AAMR: age‐adjusted mortality rate
AAPC: average annual percent change
APC: annual percent change
CDC: centers for disease control and prevention
CDC WONDER: centers for disease control and prevention‐wide‐ranging online data for epidemiologic research
CEPD: cerebral embolic protection device
CI: confidence interval
CMR: crude mortality rate
CT: computed tomography.
DALYS: disability‐adjusted life years.
ICD‐10: International Classification of Diseases, Tenth Revision
ICD‐8: International Classification of Diseases, Eight Revision
ICD‐9: International Classification of Diseases, Ninth Revision
MRI: magnetic resonance imaging
NCHS: National Center for Health Statistics
NH: non‐Hispanic
STROBE: strengthening the reporting of observational studies in epidemiology
tPA: tissue plasminogen activator.
US: United States.

## 1 Introduction

Stroke remains one of the leading causes of mortality and long‐term disability worldwide, with ischemic stroke accounting for nearly 85% of all cases and approximately 5.5 million deaths annually (Mukherjee and Patil 2011). An ischemic stroke occurs when an obstruction, commonly thrombotic or embolic blocks, impedes blood flow in a cerebral artery, depriving brain tissue of oxygen and nutrients. The Centers for Disease Control and Prevention (CDC) report that 795,000 people in the United States (US) experience a new or recurrent stroke each year, of which about 610,000 are first attacks (Stroke Facts | Stroke | CDC n.d., Cheng et al. 2025). Despite considerable advances in prevention, acute treatment, and rehabilitation, ischemic stroke continues to place a profound burden on patients, families and health systems. Importantly, several clinical and laboratory variables, including premorbid cardiovascular therapy, inflammatory markers, and acute neurological severity, have been identified as predictive factors for post‐stroke mortality, as demonstrated in large cohort studies such as the GIFA investigations. These demonstrate that factors such as prior use of antiplatelet or antihypertensive agents, elevated white blood cell count, hyperglycemia on admission, and greater stroke severity significantly influence survival trajectories and functional outcomes following acute ischemic stroke (Tuttolomondo et al. 2011).

Since 1968, epidemiological studies have documented substantial changes in the incidence, risk factors, management, and outcomes of ischemic stroke. The late 20th century witnessed declining age‐adjusted stroke mortality rates in many high‐income countries largely attributed to improved hypertension detection and control, reduced smoking prevalence, and advances in acute medical care (Lackland et al. 2013). However, these declines have not been uniform across populations. Persistent disparities in stroke incidence and outcomes remain evident, particularly among racial and ethnic minorities, rural communities, and socio‐economically disadvantaged groups. These inequities highlight the ongoing need for targeted prevention strategies and equitable access to evidence based care. Furthermore, acute ischemic stroke is a highly heterogenous disease, and meaningful interpretation of clinical outcomes requires distinguishing between major ischemic stroke subtypes such as cardioembolic stroke, lacunar infarction, atherothrombotic infarction, essential cerebral infarction, and strokes of unusual etiology, which differ substantially in pathophysiology, risk factor profiles, severity, and prognosis (Gasull and Arboix 2022).

Public health surveillance data have been critical in identifying trends over time. The Centers for Disease Control and Prevention Wide‐Ranging Online Data for Epidemiologic Research (CDC‐WONDER) and other agencies have documented a decline in stroke mortality over recent decades, yet these gains are threatened by rising cardio metabolic disease and widening health disparities (CDC WONDER n.d.). Moreover, geographic variability remains a critical challenge, with higher stroke mortality rates observed in the southeastern US, often referred to as the stroke belt. Addressing these disparities requires both population wide interventions, such as improving access to preventive care, and community specific strategies to mitigate social determinants of health. As we review evidence from 1968 to 2022 it is essential to contextualize ischemic stroke not only as a clinical entity but also as a reflection of broader public health challenges. Evaluating historical patterns, treatment innovations, and persistent disparities can inform future strategies to reduce stroke incidence, promote acute and long‐term outcomes, and minimize the global burden of disability. Considering the heterogeneity of stroke and the influence of clinical predictors on mortality, examining long‐term trends provides an opportunity to better understand how shifting population health profiles, treatment practices, and subtype distributions shape outcomes over time.

## 2 Methods

### 2.1 Data Source and Data Extraction

In this descriptive study, we used the data provided by the National Center for Health Statistics (NCHS), which was made available through the CDC‐WONDER database, to analyze annual mortality trends from 1968–2023. Death certificates of US residents are annually updated on this database. The study was conducted and strictly adhered to the Strengthening the Reporting of Observational Studies in Epidemiology (STROBE) guidelines (von Elm et al. 2008). We used the final multiple cause‐of‐death public‐use records and International Classification of Diseases codes from the 8th, 9th, and 10th revisions, corresponding to different calendar periods. ICD‐8 codes 433–434 were applied for deaths occurring between 1968 and 1978, ICD‐9 codes 433–434 for 1979 to 1998, and ICD‐10 code I63 for deaths from 1999 onward. These codes were also consistent with prior studies on stroke (Waqas et al. 2025).

Our analysis focused on death certificates from the database, capturing cases where ischemic stroke was listed as a primary cause of death. The study did not require institutional review board (IRB) approval because we used a publicly accessible, de‐identified dataset provided by the government that had no connection with human subjects.

### 2.2 Data Abstraction

Information on population size, deaths, and percentage of total deaths was retrieved. The dataset was thoroughly analyzed for a wide range of demographic variables, that is, overall years, sex, race/ethnicity, and age group. Race/ethnicity groups were delineated as non‐Hispanic (NH) White, and NH Black or African American. For age stratification, age was divided into the following categories: 25–44 years, 45–64 years, and ≥65 years.

### 2.3 Statistical Analysis

To determine national trends and disparities in mortality for cerebrovascular accidents (i.e., ischemic stroke), we measured the crude (CMRs), age‐adjusted mortality rates (AAMRs), and 95% confidence intervals (CIs) were calculated per 100,000 people from 1968 to 2023. CMR for that year was calculated by dividing the number of fatalities or casualties from ischemic stroke by the total US population for the given year. To enable comparisons across various demographic groupings and historical periods, age adjustment was conducted using the 2000 US standard population as a reference (Anderson and Rosenberg 1998). Mortality rates were estimated based on year, sex, race/ethnicity, and age groups.

The Joinpoint Regression Program (Version 5.3.0, National Cancer Institute) was used to analyze significant changes in mortality over time (Joinpoint Regression Program n.d., National Cancer Institute (NCI) n.d.). This regression method identifies statistically significant changes in trends by fitting log‐linear models. This approach detected instances when a statistically significant change in trend occurred, allowing for the calculation of annual percent changes (APCs) and average annual percent changes (AAPCs) for the overall trend for each demographic variable, in addition to their 95% CIs. APCs and AAPCs were considered significant if the associated 95% CIs did not include zero and had a *p*‐value of less than 0.05.

## 3 Results

### 3.1 Annual Trends in Mortality Rates From 1968 to 2023 in the US

**FIGURE 1:**
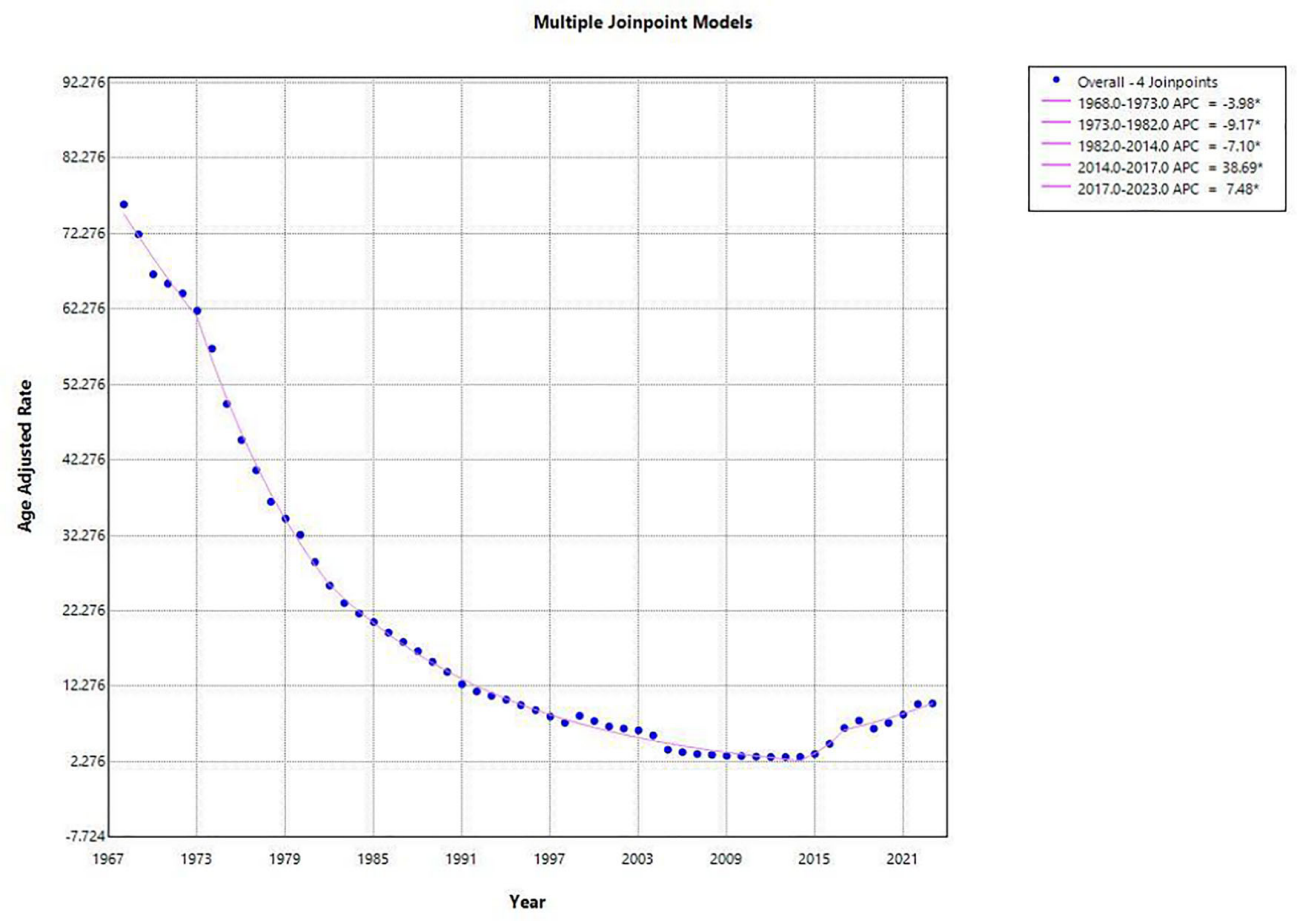
Graphical representation showing annual trends in AAMR for ischemic stroke in the United States, 1968–2023.

From 1968 to 2023, a total of 1,363,668 deaths occurred from ischemic stroke. The AAMR for ischemic stroke in the US declined substantially from 76.2 to 10.0 per 100,000 population, reflecting an overall decrease of 86.9% over the 55‐year period (AAPC: −3.59 [95% CI: ‐5.11 to −2.06, *p* = 0.000006]), indicating a sustained long‐term reduction as shown in Figure 1. The AAMR decreased significantly from 1968 to 1973 (APC: −3.98 [95% CI: −6.42 to −1.46]), followed by a steeper decline from 1973 to 1982 (APC: −9.17 [95% CI: −10.48 to −7.84]). A consistent reduction persisted from 1982 to 2014 (APC: −7.10 [95% CI: −7.43 to −6.78]). However, from 2014 to 2017, mortality rates increased sharply (APC: 38.69 [95% CI: 4.50 to 84.06]), with the upward trend continuing from 2017 to 2023 (APC: 7.48 [95% CI: 4.19 to 10.87]) (Supplementary Table 1).

### 3.2 Sex‐Specific Trends in Mortality Rates From 1968 to 2023 in the US

**FIGURE 2:**
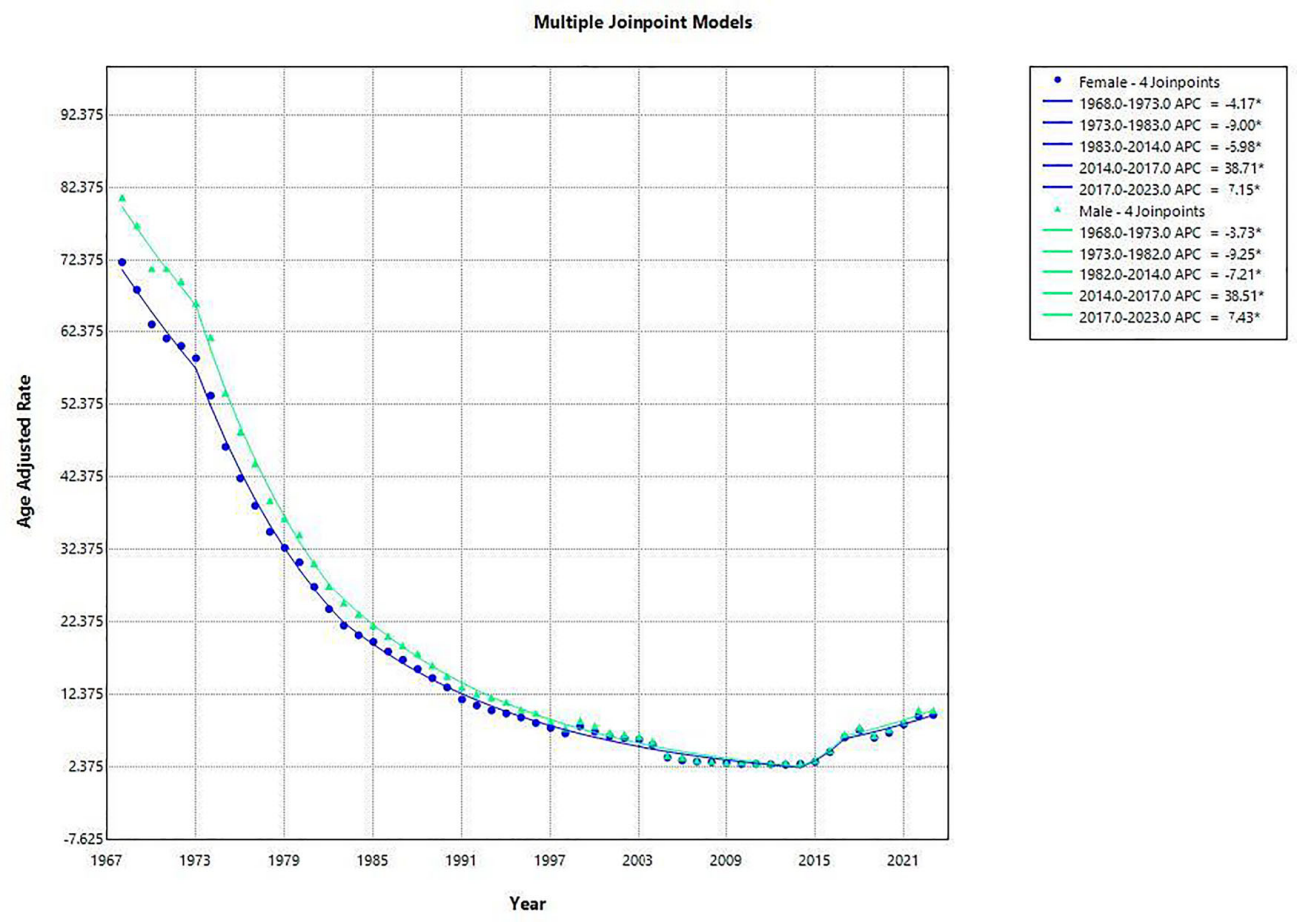
Graphical representation showing sex‐specific trends in AAMR for ischemic stroke in the United States, 1968–2023.

Among females, a total of 788964 deaths occurred due to ischemic stroke. The AAMR demonstrated a substantial decline from 72.17 per 100,000 in 1968 to 9.61 in 2023. Five segments were identified: The AAMR declined from 1968 to 1973 (APC: −4.17 [95% CI: −6.69 to −1.59, *p* = 0.002]). A steeper decline followed between 1973 and 1983 (APC: −9.00% [95% CI: −10.13 to ‐7.85, *p* < 0.000001]). The decreasing trend continued, though at a slower rate between 1983 and 2014 (APC: −6.98 [95% CI: −7.32 to ‐6.63]). However, the trend reversed with a significant increase in AAMR during 2014 to 2017 (APC: 38.71 [95% CI: 1.80 to 89.01]). The upward trend persisted between 2017 and 2023 (APC: 7.15 [95% CI: 3.76 to 10.66]), as depicted in Figure 2. The average annual percent change (AAPC) across the full study period was −3.57% (95% CI: −5.22 to −1.90, *p* < 0.001).

Among males, ischemic stroke caused 574704 deaths over this period. The AAMR decreased from 81.1 per 100,000 in 1968 to 10.26 in 2023. Five distinct segments were identified: From 1968 to 1973, AAMRs declined initially from 1968 to 1973 (APC: ‐3.73 [95% CI: −6.17 to −1.23]), followed by a sharper drop between 1973 and 1982 (APC: 9.25 [95% CI: −10.61 to −7.86]). Between 1982 and 2014, the rate continued to decline during the period 1982 to 2014 (APC: −7.21 [95% CI: −7.54 to −6.89]). From 2014 to 2017, this was followed by a significant increase from 2014 to 2017 (APC: 38.51 [95% CI: 4.23 to 84.08]), which continued from 2017 to 2023 (APC: 7.43 [95% CI: 4.26 to 10.69]). The overall AAPC for males over the full range was ‐3.66 (95% CI: −5.18 to −2.12, *p* < 0.00001) (Supplementary Table 2).

### 3.3 Race‐Specific Trends in Mortality Rates From 1968 to 2023 in the US

From 1968 to 2023, ischemic stroke accounted for 141,991 deaths among Black or African Americans and 1,203,947 deaths among whites.

**FIGURE 3:**
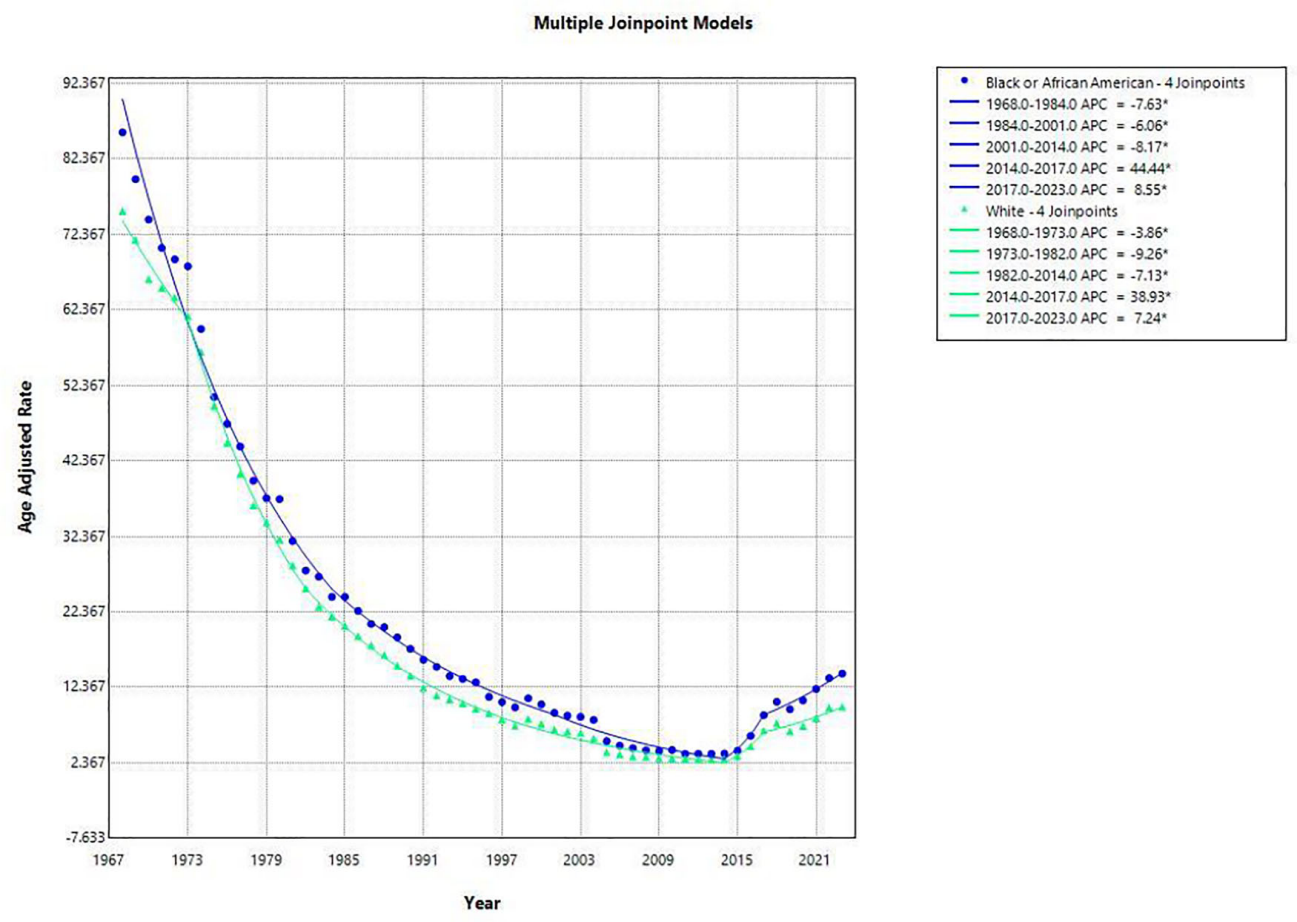
Graphical representation showing racial trends in AAMR for ischemic stroke in the United States, 1968–2023.

From 1968 to 2023, the AAMR for ischemic stroke among the Black or African American population decreased from 85.9 deaths per 100,000 in 1968 to 14.2 in 2023, representing an overall reduction of approximately 83.5%. Between 1968 and 1984, the AAMR declined significantly with an (APC: −7.63 [95% CI: −8.17 to −7.08]). The decline continued from 1984 to 2001 (APC: −6.06 [95% CI: −6.84 to −5.27]), and further from 2001 to 2014 with a sharper (APC: −8.17 [95% CI: −9.84 to −6.48]). A reversal was observed, where the rate increased rapidly in between 2014 to 2017 (APC: 44.44 [95% CI: 8.84 to 91.69]). The upward trend persisted but at a slower rate during the period 2017 to 2023 [APC: 8.55 (95% CI: 5.42 to 11.78)] as shown in Figure 3. Over the full period, the AAPC was −3.30 (95% CI: −4.85 to −1.72; *p* < 0.0001), indicating a sustained long‐term decline despite recent increases.

Among the white population, ischemic stroke mortality declined from 75.5 deaths per 100,000 in 1968 to 9.8 in 2023, a total decrease of about 87.0%. Initially the rate declined during 1968 to 1973 (APC: −3.86% [95% CI: −6.33 to −1.32]). A steeper decline occurred from 1973 to 1982 (APC: −9.26 [95% CI: −10.58 to −7.91]), continuing between 1982 and 2014 (APC: −7.13 [95% CI: −7.45 to −6.81]). A sharp increase was observed from 2014 to 2017 (APC: 38.93 [95% CI: 3.13 to 87.17]), followed by a continued increase from 2017 to 2023 (APC: 7.24 [95% CI: 3.80 to 10.80]). The AAPC over the entire study period was −3.63 (95% CI: −5.21to −2.01; *p* < 0.0001), confirming a strong overall decline (Supplementary Table 3).

### 3.4 Age‐Specific Trends in Mortality Rates From 1968 to 2023 in the US

**FIGURE 4:**
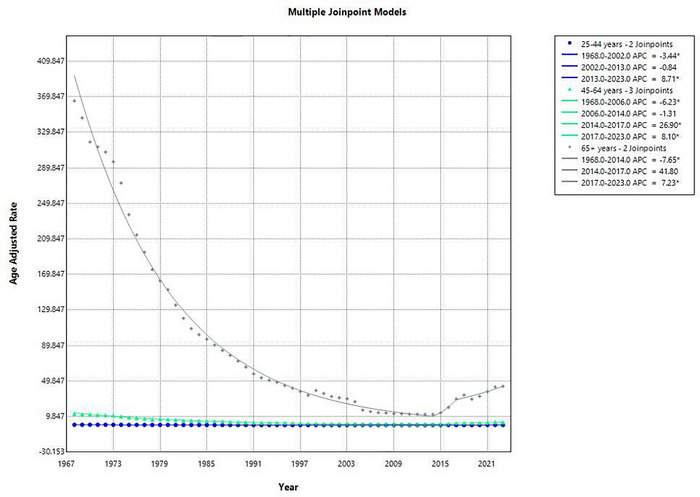
Graphical representation showing age group trends in AAMR for ischemic stroke in the United States, 1968–2023.

From 1968 to 2023, a total of 12,488 deaths occurred among adults aged 25–44 years. In 1968, the AAMR was 0.63 per 100,000, which declined to 0.42 in 2023, with an overall decreasing trend over the 55‐year period (AAPC: −0.81 [95% CI: −1.33 to −0.28], *p* = 0.0026). A sharp decline was observed from 1968 to 2002 (APC: −3.44 [95% CI: −3.69 to −3.19]), followed by a non‐significant plateau between 2002 and 2013 (APC: −0.84 [95% CI: −2.98 to 1.35]). However, a marked rise occurred from 2013 to 2023 (APC: 8.71 [95% CI: 7.04 to 10.41]), indicating a recent reversal in this age group's mortality trend. Refer to Figure 4.

Among adults aged 45–64 years, 132,317 deaths occurred due to ischemic stroke. The AAMR declined from 13.08 per 100,000 in 1968 to 3.57 in 2023, reflecting an overall decrease (AAPC: −2.46 [95% CI: ‐3.78 to −1.11], *p* = 0.0004). The mortality rate decreased significantly from 1968 to 2006 (APC: −6.23 [95% CI: −6.40 to −6.05]), remained statistically unchanged from 2006 to 2014 (APC: −1.31 [95% CI: −5.01 to 2.53]), then spiked sharply from 2014 to 2017 (APC: 26.90 [95% CI: 0.85 to 59.68]), and continued increasing from 2017 to 2023 (APC: 8.10 [95% CI: 5.36 to 10.91]).

A total of 1218863 deaths were recorded in older adults aged 65 years and above, indicating the most substantial mortality burden, though the AAMR showed a strong overall decline from 365.21 in 1968 to 44.02 in 2023 (AAPC: −3.91 [95% CI: −5.94 to ‐1.83], *p* = 0.0003). The period from 1968 to 2014 saw a significant and steady decrease (APC: −7.65 [95% CI: −7.84 to −7.46]), followed by a non‐significant uptick between 2014 and 2017 (APC: 41.80 [95% CI: −4.26 to 110.01]), and a significant increase from 2017 to 2023 (APC: 7.23 [95% CI: 2.92 to 11.72]) (Supplementary Table 4).

## 4 Discussion

The findings from this study reveal that while ischemic stroke mortality in the US significantly declined from 1968 to 2014, a reversal in this trend emerged after 2014. The rise in obesity, diabetes, and substance use is a broad explanation for this shift, as these factors are linked to an increased risk of stroke. However, it is essential to recognize that these risk factors do not affect all subgroups equally. The impact of these conditions varies considerably depending on age, geographic location, and other demographic factors. Obesity, diabetes, and substance use have likely contributed to the resurgence of ischemic stroke mortality across all demographic stratifications. Yet, their effects appear to disproportionately affect certain populations. For example, younger adults (aged 25–44 years) have seen a sharper rise in obesity and diabetes prevalence in recent years, which may explain the reversal in mortality trends within this age group. The combination of these emerging risk factors, along with the challenges of accessing timely medical care, may exacerbate stroke risk in younger adults, who might not typically be seen as high‐risk populations. Additionally, younger adults may face greater barriers in controlling these comorbidities due to lifestyle factors, including poor diet, sedentary behavior, and limited health literacy, further compounding the effects of rising obesity and diabetes rates.

Historically, the substantial decline in stroke mortality observed during the late 20th century can be attributed in part to major diagnostic and therapeutic breakthroughs. The introduction of computed tomography (CT) in the 1970s revolutionized stroke diagnosis by distinguishing ischemic from hemorrhagic events, while the subsequent adoption of magnetic resonance imaging (MRI) enhanced the ability to detect early ischemic changes (McCollough and Rajiah 2023, Kumar et al. 2024). Therapeutically, the approval of intravenous tissue plasminogen activator (tPA) in the 1990s marked a major milestone (Kim 2019). More recently, endovascular thrombectomy has emerged as a standard of care for patients with large vessel occlusion, significantly improving functional outcomes when administered within an appropriate time window (Makkawi et al. 2024). In parallel, sentinel cerebral embolic protection devices (CEPDs) have been developed to minimize the risk of distal embolization during such procedures, offering an additional safeguard against periprocedural ischemic complications and further refining the safety profile of contemporary endovascular therapy (Kapadia et al. 2022). In addition, the changing epidemiology of incident strokes, with a progressively higher proportion of minor and non‐disabling ischemic events, may partly explain the earlier decline in mortality, as these patients are more likely to survive due to improved diagnostic sensitivity and greater adherence to increasingly effective antithrombotic and secondary prevention therapies (Benjamin et al. 2018, Ay et al. 2010).

Despite these advances, the global burden of ischemic stroke remains substantial. It continues to rank among the top five causes of death in the US and is a leading contributor to disability‐adjusted life years (DALYS) worldwide (Feigin et al. 2025). Global analyses by the GBD 2019 Stroke Collaborators confirm that stroke remains a leading cause of death and disability worldwide, with rising incidence in low‐ and middle‐income regions and a plateauing decline in high‐income nations (GBD 2019 Stroke Collaborators 2021). The absolute number of stroke cases is rising, driven by population aging, increasing prevalence of atrial fibrillation diabetes, obesity, and poor lifestyle and diet. Furthermore, recurrent strokes contribute significantly to long term disability, highlighting gaps in secondary prevention strategies, including anticoagulation for atrial fibrillation and control of vascular risk factors.

The rise in obesity, diabetes, and substance use has likely contributed to the resurgence of ischemic stroke mortality across all demographic stratifications. Yet, their effects appear to disproportionately affect certain populations. For example, younger adults (aged 25–44 years) have seen a sharper rise in obesity and diabetes prevalence in recent years, which may explain the reversal in mortality trends within this age group. The combination of these emerging risk factors, along with the challenges of accessing timely medical care, may exacerbate stroke risk in younger adults, who might not typically be seen as high‐risk populations (Vlajinac et al. 1994, Melby 2017, Centers for Disease Control and Prevention, National Center for Health Statistics 2000). Additionally, younger adults may face greater barriers in controlling these comorbidities due to lifestyle factors, including poor diet, sedentary behavior, and limited health literacy, further compounding the effects of rising obesity and diabetes rates. A recent study by Lim et al. (2025) supports these findings, noting that rising mortality rates in non‐metropolitan areas are closely linked with increasing obesity and diabetes prevalence.

The sudden decline of the AAMRs due to cerebrovascular diseases, with an overall 86.9 percent reduction (76.2 to 10.0 per 100,000 population), constitutes one of the biggest public health milestones in cerebrovascular disease prevention. Despite this achievement, the trends in ischemic stroke mortality began to reverse after 2014 (Murray and Lopez 1996). Comparable shifts in other cardiovascular domains have been documented, including a recent rise in arrhythmia‐related deaths in the United States after decades of decline, suggesting a shared cardiometabolic and lifestyle‐driven epidemiologic transition (Mhanna et al. 2025). Men consistently exhibited higher AAMRs than women, and both sexes have experienced increasing mortality rates in recent years. The rise in mortality, particularly among men, is likely attributed to the growing burden of cardiometabolic risk factors, including obesity, hypertension, and diabetes (La Vecchia et al. 1993). Furthermore, women, who are disproportionately affected by conditions like atrial fibrillation and diabetes, face additional challenges related to socioeconomic factors. Women from lower socioeconomic backgrounds often experience greater difficulty in managing these comorbidities due to barriers like healthcare access, medication affordability, and lack of preventive care. These trends suggest that while both sexes have benefited from improvements in stroke care and prevention, the recent uptick in mortality reflects not only biological risk factors but also persistent healthcare disparities (Rastenyte et al. 1992, Sommers et al. 2015, Bailey et al. 2015).

Racial and ethnic disparities remain a significant challenge in addressing ischemic stroke mortality. The study observed that Black or African American populations have historically experienced higher stroke mortality rates compared to white populations, and the reversal in trends post‐2014 has been more pronounced among Black individuals. These populations continue to experience persistent healthcare disparities, including limited access to high‐quality stroke care, lower rates of preventive services, and inadequate treatment adherence. Structural inequalities, such as limited insurance coverage, fewer specialized stroke care facilities, and systemic racism within the healthcare system, exacerbate these disparities, leading to poorer outcomes for Black patients. Despite declines in mortality over time, Black adults still have a greater burden of ischemic stroke mortality, and the scale of the post‐2014 rise was particularly notable during the period from 2014 to 2017, when the AAMRs for Black individuals increased by 44.4%. These findings are consistent with the conclusion that racial disparities in healthcare access and delivery are critical determinants of health outcomes (Bailey et al. 2015, Vasan et al. 2019, Kernan et al. 2014).

Geographic and socioeconomic factors further exacerbate ischemic stroke mortality, particularly in rural versus urban areas. Rural populations face unique challenges, such as limited access to specialized stroke care, fewer healthcare professionals, and long travel distances to medical facilities. These barriers often result in delayed diagnoses, longer treatment times, and poor management of stroke risk factors. In rural areas, the prevalence of obesity, diabetes, and smoking is also higher, further increasing the stroke burden. The resurgence in mortality among rural populations underscores the need for improved healthcare infrastructure in these regions, including better access to stroke prevention programs, education, and timely treatment (Lim et al. 2025, Sommers et al. 2015, Penner et al. 2017). A recent study by Ahmad et al. (2025) highlighted that the growing impact of atrial fibrillation on stroke mortality is particularly acute in non‐metropolitan regions, which suggests that geographic factors continue to contribute to the growing burden of ischemic stroke mortality (Ahmad et al. 2024).

These findings suggest that the recent increase in mortality is due to a combination of rising cardiometabolic risk factors, persistent disparities in insurance coverage and healthcare access, and the unequal distribution of specialized stroke care, especially in rural and underserved areas (Kleindorfer et al. 2021). This epidemiologic reversal underscores the urgent need to re‐evaluate national prevention strategies and address the social determinants of cardiovascular health to sustain earlier public health gains (GBD 2019 Stroke Collaborators 2021, Mhanna et al. 2025).

The major strength of this study lies in its unique five‐decade span, which provides one of the most comprehensive evaluations of long‐term ischemic stroke mortality in the US. Few analyses have captured trends over such an extended timeframe, allowing for a nuanced understanding of both the successes of the late 20th century and the reversals of the last decade. The use of the CDC's population‐based mortality dataset further enhances the reliability of the findings, as it reflects a nationally representative and robust sample size with standardized methods of death certification and ICD coding across decades. The application of joinpoint regression also allowed for precise detection of temporal shifts, providing important insights into inflection points where trends accelerated, plateaued, or reversed.

Future research should aim to examine how evolving stroke phenotypes, including the rising proportion of minor strokes, interact with treatment patterns and long‐term outcomes at the population level. Linking national mortality data with clinical registries could provide deeper insight into risk factor trajectories, adherence to antithrombotic therapies, and the contribution of emerging metabolic and lifestyle determinants to recent mortality reversals. Additionally, longitudinal studies that incorporate social determinants of health, regional care disparities, and treatment access gaps would help identify modifiable intervention points to prevent further increases in ischemic stroke mortality.

Nonetheless, several limitations must be acknowledged. First, this study relied on the CDC's mortality dataset, which is dependent on the accuracy of death certificates. Misclassification of cause of death and changes in ICD coding practices over time could introduce bias, although the persistence of trends across sex, race, and age groups suggests genuine epidemiological shifts rather than artifacts. Second, as an observational study, the analysis cannot establish causal relationships between policy changes, risk factor trends, and observed mortality patterns. Third, the dataset lacks detailed clinical information such as comorbidities, medication adherence, stroke subtypes, treatment modalities, and risk factor control levels, all of which are critical to fully understanding mortality dynamics. Fourth, the analysis did not incorporate social determinants of health, including income, education, geographic access to care, and structural inequities, which play a pivotal role in shaping both risk factor burden and outcomes. Finally, future research should conduct sensitivity analyses to test the robustness of these findings, including adjustments for misclassification bias, state‐level variation in healthcare access, and the impact of emerging risk factors such as obesity and substance use on stroke mortality.

## 5 Conclusion

It is concluded that ischemic stroke mortality in the US declined substantially between 1968 and 2014, reflecting major advances in cardiovascular prevention, risk factor control, and acute stroke management. However, since the mid‐2010s, this trend has reversed, with significant increases observed across sex, racial, and age groups, most notably among younger adults and Black populations. These findings highlight the urgent need for renewed, targeted public health strategies and interventions aimed at controlling modifiable vascular risk factors, reducing healthcare disparities, and improving access to timely stroke care. Without decisive action, the hard‐earned gains of the past five decades risk being eroded.

## Author Contributions

**Rameez Qasim**: conceptualization, data curation, and resources. **Laiba Muzammil**: formal analysis, investigation, and writing – original draft. **Bilal Qammar**: formal analysis, and writing – review and editing. **Areej Dar, Laiba Sultan**: methodology and writing – original draft. **Mubashir Raza, Umama Alam, Muhammad Khalid Afridi, Saad Ahmed Waqas, and Hasibullah Aminpoor**: writing – review and editing. **Raheel Ahmed**: supervision and validation. The final version of this manuscript has been read and approved by all the authors.

## Funding

The authors have nothing to report.

## Conflicts of Interest

The authors declare no conflicts of interest.

## Supporting information




**Supplementary Tables**: brb371177‐sup‐0001‐Tables.docx

## Data Availability

The data analyzed in this study were obtained from the CDC WONDER database, which provides access to a wide range of public health datasets. The mortality data used in this analysis are publicly available and can be accessed through the CDC WONDER website at: https://wonder.cdc.gov/. Any additional data that support the findings of this study are available from the corresponding author upon reasonable request.
