# Supplementary material for: Five‐Decade Mortality Trends in Ischemic Stroke in the United States: A CDC WONDER Analysis
Source: Brain Behav. 2025 Dec 31;16(1):e71177. doi: 10.1002/brb3.71177 (PMC12755966; doi:10.1002/brb3.71177)
Supplement: Supplementary file 1 — Supplementary Tables: brb371177‐sup‐0001‐Tables.docx [file BRB3-16-e71177-s001.docx]

**SUPPLEMENTARY TABLES**

| **Period** | **AAMR Start** | **AAMR End** | **APC (%)** | **95% CI** | **p-value** |
| --- | --- | --- | --- | --- | --- |
| 1968–1973 | 76.20 | 63.14 | –3.98 | –6.42 to –1.46 | 0.002 |
| 1973–1982 | 63.14 | 27.67 | –9.17 | –10.48 to –7.84 | <0.000001 |
| 1982–2014 | 27.67 | 6.88 | –7.10 | –7.43 to –6.78 | <0.000001 |
| 2014–2017 | 6.88 | 9.74 | 38.69 | 4.50 to 84.06 | 0.021 |
| 2017–2023 | 9.74 | 10.00 | 7.48 | 4.19 to 10.87 | <0.0001 |
| **Overall (AAPC)** | — | — | –3.59 | –5.11 to –2.06 | 0.000006 |

**Supplementary Table 1.** Annual Trends in Age-Adjusted Mortality Rates (AAMR) for Ischemic Stroke in the United States, 1968–2023

| **Gender** | **Period** | **AAMR Start** | **AAMR End** | **APC (%)** | **95% CI** | **p-value** | **AAPC (%)** | **95% CI** | **p-value** |
| --- | --- | --- | --- | --- | --- | --- | --- | --- | --- |
| Female | 1968–1973 | 72.17 | 58.13 | –4.17 | –6.69 to –1.59 | 0.002 | –3.57 | –5.22 to –1.90 | <0.001 |
|  | 1973–1983 | 58.13 | 25.21 | –9.00 | –10.13 to –7.85 | <0.000001 |  |  |  |
|  | 1983–2014 | 25.21 | 6.26 | –6.98 | –7.32 to –6.63 | <0.000001 |  |  |  |
|  | 2014–2017 | 6.26 | 8.69 | 38.71 | 1.80 to 89.01 | 0.038 |  |  |  |
|  | 2017–2023 | 8.69 | 9.61 | 7.15 | 3.76 to 10.66 | <0.0001 |  |  |  |
| Male | 1968–1973 | 81.10 | 67.55 | –3.73 | –6.17 to –1.23 | 0.004 | –3.66 | –5.18 to –2.12 | <0.00001 |
|  | 1973–1982 | 67.55 | 29.65 | –9.25 | –10.61 to –7.86 | <0.000001 |  |  |  |
|  | 1982–2014 | 29.65 | 7.50 | –7.21 | –7.54 to –6.89 | <0.000001 |  |  |  |
|  | 2014–2017 | 7.50 | 10.39 | 38.51 | 4.23 to 84.08 | 0.019 |  |  |  |
|  | 2017–2023 | 10.39 | 10.26 | 7.43 | 4.26 to 10.69 | <0.0001 |  |  |  |

**Supplementary Table 2.** Gender-Specific Trends in AAMR for Ischemic Stroke, United States, 1968–2023

| **Race/Ethnicity** | **Period** | **AAMR Start** | **AAMR End** | **APC (%)** | **95% CI** | **p-value** | **AAPC (%)** | **95% CI** | **p-value** |
| --- | --- | --- | --- | --- | --- | --- | --- | --- | --- |
| Black/African American | 1968–1984 | 85.90 | 48.78 | –7.63 | –8.17 to –7.08 | <0.000001 | –3.30 | –4.85 to –1.72 | <0.0001 |
|  | 1984–2001 | 48.78 | 23.92 | –6.06 | –6.84 to –5.27 | <0.000001 |  |  |  |
|  | 2001–2014 | 23.92 | 7.41 | –8.17 | –9.84 to –6.48 | <0.000001 |  |  |  |
|  | 2014–2017 | 7.41 | 10.72 | 44.44 | 8.84 to 91.69 | 0.015 |  |  |  |
|  | 2017–2023 | 10.72 | 14.20 | 8.55 | 5.42 to 11.78 | <0.0001 |  |  |  |
| White | 1968–1973 | 75.50 | 62.47 | –3.86 | –6.33 to –1.32 | 0.003 | –3.63 | –5.21 to –2.01 | <0.0001 |
|  | 1973–1982 | 62.47 | 27.28 | –9.26 | –10.58 to –7.91 | <0.000001 |  |  |  |
|  | 1982–2014 | 27.28 | 6.77 | –7.13 | –7.45 to –6.81 | <0.000001 |  |  |  |
|  | 2014–2017 | 6.77 | 9.39 | 38.93 | 3.13 to 87.17 | 0.025 |  |  |  |
|  | 2017–2023 | 9.39 | 9.80 | 7.24 | 3.80 to 10.80 | <0.0001 |  |  |  |

**Supplementary Table 3.** Race-Specific Trends in AAMR for Ischemic Stroke, United States, 1968–2023

| **Age Group (years)** | **Period** | **AAMR Start** | **AAMR End** | **APC (%)** | **95% CI** | **p-value** | **AAPC (%)** | **95% CI** | **p-value** |
| --- | --- | --- | --- | --- | --- | --- | --- | --- | --- |
| 25–44 | 1968–2002 | 0.63 | 0.34 | –3.44 | –3.69 to –3.19 | <0.000001 | –0.81 | –1.33 to –0.28 | 0.0026 |
|  | 2002–2013 | 0.34 | 0.32 | –0.84 | –2.98 to 1.35 | 0.45 |  |  |  |
|  | 2013–2023 | 0.32 | 0.42 | 8.71 | 7.04 to 10.41 | <0.0001 |  |  |  |
| 45–64 | 1968–2006 | 13.08 | 4.67 | –6.23 | –6.40 to –6.05 | <0.000001 | –2.46 | –3.78 to –1.11 | 0.0004 |
|  | 2006–2014 | 4.67 | 4.13 | –1.31 | –5.01 to 2.53 | 0.51 |  |  |  |
|  | 2014–2017 | 4.13 | 5.24 | 26.90 | 0.85 to 59.68 | 0.041 |  |  |  |
|  | 2017–2023 | 5.24 | 3.57 | 8.10 | 5.36 to 10.91 | <0.0001 |  |  |  |
| ≥65 | 1968–2014 | 365.21 | 41.18 | –7.65 | –7.84 to –7.46 | <0.000001 | –3.91 | –5.94 to –1.83 | 0.0003 |
|  | 2014–2017 | 41.18 | 58.37 | 41.80 | –4.26 to 110.01 | 0.09 |  |  |  |
|  | 2017–2023 | 58.37 | 44.02 | 7.23 | 2.92 to 11.72 | 0.001 |  |  |  |

**Supplementary Table 4.** Age-Specific Trends in AAMR for Ischemic Stroke, United States, 1968–2023
